# Hydrocortisone administration was associated with improved survival in Japanese patients with cardiac arrest

**DOI:** 10.1038/s41598-017-17686-3

**Published:** 2017-12-20

**Authors:** Takahiro Niimura, Yoshito Zamami, Toshihiro Koyama, Yuki Izawa-Ishizawa, Masashi Miyake, Tadashi Koga, Keisaku Harada, Ayako Ohshima, Toru Imai, Yutaka Kondo, Masaki Imanishi, Kenshi Takechi, Keijo Fukushima, Yuya Horinouchi, Yasumasa Ikeda, Hiromichi Fujino, Koichiro Tsuchiya, Toshiaki Tamaki, Shiro Hinotsu, Mitsunobu R. Kano, Keisuke Ishizawa

**Affiliations:** 1 Department of Clinical Pharmacology and Therapeutics, Institute of Biomedical Sciences, Tokushima University Graduate School, 3-18-15 Kuramoto-cho, Tokushima, 770-8503 Japan; 2 Department of Pharmacy, Tokushima University Hospital, 2-50-1 kuramoto-cho, Tokushima, 770-8503 Japan; 3 Department of Emergency Pharmaceutical Science, Graduate School of Medicine, Dentistry and Pharmaceutical Sciences, Okayama University, 1-1-1 Tsushima-naka, Okayama, 700-8530 Japan; 4 Department of Clinical Pharmacy, Graduate School of Medicine, Dentistry and Pharmaceutical Sciences, Okayama University, 2-5-1 Shikata-cho, Okayama, 700-8558 Japan; 5 Department of Pharmacology, Institute of Biomedical Sciences, Tokushima University Graduate School, 3-18-15 Kuramoto-cho, Tokushima, 770-8503 Japan; 6 Research Support Department, Drug Safety Research Laboratories, Pharmacokinetics and Bioanalysis Center, Shin Nippon Biomedical Laboratories, Ltd, 2438 Miyanoura, Kagoshima, 891-1394 Japan; 7 Department of Pharmacy, Kitakyushu City Yahata Hospital, 4-18-1 Nishihonmachi, Kitakyushu-shi, Fukuoka 805-8534 Japan; 8 Department of Pharmaceutical Biomedicine, Graduate School of Medicine, Dentistry and Pharmaceutical Sciences, Okayama University, 1-1-1 Tsushima-naka, Okayama, 700-8530 Japan; 9 Department of Pharmacy, Nihon University Itabashi Hospital, 30-1 Oyaguchi-Kami Machi, Tokyo, 173-8610 Japan; 10 Division of Acute Care Surgery, Trauma, and Surgical Critical Care, Department of Surgery, Beth Israel Deaconess Medical Center, Harvard Medical School, 330, Brookline Avenue, Boston, MA 02215 USA; 11 Clinical Trial Center for Developmental Therapeutics, Tokushima University Hospital, 2-50-1 Kuramoto-cho, Tokushima, 770-8503 Japan; 12 Department of Molecular Pharmacology, Graduate School of Pharmaceutical Sciences & Graduate School of Biomedical Sciences, Tokushima University, 1-78-1 Shinkura-cho, Tokushima, 770-8501 Japan; 13 Department of Medical Pharmacology, Institute of Biomedical Sciences, Tokushima University Graduate School, 1–78–1 Sho-machi, Tokushima, 770-8505 Japan; 14 Center for Innovative Clinical Medicine, Okayama University Hospital, 2-5-1 Shikata-cho, Okayama, 700-8558 Japan

## Abstract

There are few reports on hydrocortisone administration after cardiac arrest, and those that have been published included few subjects. This study aimed to evaluate the effect of hydrocortisone administration on the outcomes of patients who experienced cardiac arrest. We investigated the survival discharge rates and the length of hospital stay from cardiac arrest to discharge, stratified by use of hydrocortisone, using a Japanese health-insurance claims dataset that covers approximately 2% of the Japanese population. The study included the data of 2233 subjects who experienced either in-hospital or out-of-hospital cardiac arrest between January 2005 and May 2014. These patients were divided into two groups, based on the administration of hydrocortisone. We adjusted the baseline characteristics, medical treatment, and drug administration data of the two groups using propensity scores obtained via the inverse probability of treatment weighted method. The hydrocortisone group had a significantly higher survival discharge rate (13/61 [21.1%] vs. 240/2172 [11.0%], adjusted odds ratio: 4.2, 95% CI: 1.60–10.98, *p* = 0.004). In addition, the administration of hydrocortisone was independent predictor of survival to discharge (hazard ratio: 4.6, *p* < 0.001). The results demonstrate a correlation between hydrocortisone administration and the high rates of survival to discharge.

## Introduction

Cardiac arrest is defined as “the cessation of cardiac mechanical activity, as confirmed by the absence of signs of circulation”^1^. Approximately 356,000 out-of-hospital cardiac arrests (OHCAs) occurred from June 2014 through May 2015 in the United States^2^. In Japan, approximately 123,000 OHCAs occurred in 2015 and, given the aging of Japanese society, this number is expected to increase^3^.

Although the prognosis after cardiac arrest has improved, the rate of surviving to hospital discharge remains < 30%^4,5^. Moreover, there is a high frequency of post-resuscitation syndrome in patients who achieve return of spontaneous circulation (ROSC), and the proportion of patients able to return to normal life is extremely low^6^.

Several studies have reported that administration of glucocorticoids during and after resuscitation results in improved prognosis for cardiac arrest patients. Mentzelopoulos *et al*. reported that the rate of survival to discharge and neurological outcomes were improved by using vasopressin-steroids-epinephrine combination therapy during resuscitation and hydrocortisone for post-resuscitation shock^7,8^. In addition, Tsai *et al*. reported that using glucocorticoids during resuscitation improves the survival discharge rate, using data from the Taiwan National Health Insurance Research Database (NHIRD)^9^.

However, few reports have demonstrated the effectiveness of hydrocortisone administration during and after resuscitation. Tsai *et al*. conducted a prospective, nonrandomized, open-label clinical trial to examine the effect of hydrocortisone on OHCA outcomes^10^. They found no significant difference between the hydrocortisone and non-hydrocortisone groups in terms of rates of survival to discharge. Donnino *et al*. reported that hydrocortisone administration did not contribute to the reversal of shock, to improved neurological outcomes, or to improved rates of survival to discharge in a randomized, double-blind trial of patients with post-resuscitation shock^11^. These studies were conducted in a restricted number of medical institutions and included 100 study subjects or fewer. Glucocorticoid supplementation during and after cardiopulmonary resuscitation might confer a benefit in terms of survival to discharge, but the overall effectiveness of hydrocortisone administration in cardiac arrest is inconclusive. It is, thus, necessary to examine the effectiveness of hydrocortisone administration in various medical facilities.

Hence, in the present study, we investigated the rate of survival to discharge among patients who experienced cardiac arrest and received hydrocortisone by analyzing health-insurance claims data owned by the Japan Medical Data Center (JMDC).

## Results

### Baseline characteristics of patients

Of the 2233 patients included in this study, 61 (2.7%) were treated with hydrocortisone and 2172 (97.3%) were not. The baseline characteristics of both groups are shown in Table 1. Significant differences were observed between the hydrocortisone and non-hydrocortisone groups, respectively, in terms of the proportion of patients who achieved ROSC (25% vs. 8%, *p* < 0.001); the proportion with chronic lung disease (46% vs. 26%, *p* < 0.001) or cancer (57% vs. 39%, *p* = 0.003); the proportion of patients who received vasopressin (8% vs. 1%, *p* < 0.001), methylprednisolone (26% vs. 4%, *p* < 0.001), dopamine (64% vs. 22%, *p* < 0.001), noradrenaline (norepinephrine) (44% vs. 14%, *p* < 0.001), and lidocaine (20% vs. 6%, *p* < 0.001); the mean dosage of adrenalin (epinephrine) administered (5.63 mg vs. 2.26 mg, *p* < 0.001); tracheal intubation (51% vs. 33%, *p* = 0.004); artificial respiration (70% vs. 43%, *p* < 0.001); and hypothermia therapy (7% vs. 1%, *p* = 0.011). The groups were comparable in terms of prevalence of comorbidities. More medicines and treatments related to resuscitation were used in the hydrocortisone group than were used in the non-hydrocortisone group. The rate of survival to discharge was 21% (*n* = 13) in the hydrocortisone group and 11% (*n* = 240) in the non-hydrocortisone group.

**Table 1:** Baseline characteristics of patients in the hydrocortisone and non-hydrocortisone groups.

	Hydrocortisone group (n = 61)	Non-hydrocortisone group (n = 2172)	**Total (n=2233)**	*p*-value	Standardized mean difference
Age (years), mean ± SD	51.43 ± 14.17	51.30 ± 13.19	51.29 ± 13.21	0.835^3^	0.01
Male sex, n (%)	38 (62)	1586 (73)	1624 (73)	0.064^1^	0.24
OHCA, n (%)	6 (10)	325 (15)	331 (15)	0.360^1^	0.14
ROSC, n (%)	15 (25)	182 (8)	197 (9)	<0.001^1^	0.57
Comorbidity, n (%)					
Ischaemic heart disease	17 (28)	762 (35)	779 (35)	0.244^1^	0.15
Heart failure	18 (30)	797 (37)	815 (36)	0.250^1^	0.15
Chronic lung disease	28 (46)	572 (26)	600 (27)	<0.001^1^	0.44
Hypertension	33 (54)	1269 (58)	1302 (58)	0.499^1^	0.09
Diabetes	23 (38)	949 (44)	972 (44)	0.352^1^	0.12
Cerebrovascular disease	10 (16)	465 (21)	475 (21)	0.345^1^	0.12
Renal disease	13 (21)	297 (14)	310 (14)	0.089^1^	0.22
Liver disease	25 (41)	642 (30)	667 (30)	0.054^1^	0.25
Adrenal disease	0 (0)	16 (1)	16 (1)	1.000^2^	0.09
Hyperlipidaemia	15 (25)	579 (27)	594 (27)	0.719^1^	0.05
Cancer	35 (57)	840 (39)	875 (39)	0.003^1^	0.38
Drugs administered, n (%)					
Vasopressin	5 (8)	13 (1)	18 (1)	<0.001^2^	0.85
Methylprednisolone	16 (26)	80 (4)	96 (4)	<0.001^1^	1.11
Dopamine	39 (64)	469 (22)	508 (23)	<0.001^1^	1.01
Noradrenaline	27 (44)	300 (14)	327 (15)	<0.001^1^	0.86
Amiodarone	6 (10)	103 (5)	109 (5)	0.119^1^	0.24
Nifecarant	2 (3)	28 (1)	30 (1)	0.197^2^	0.17
Lidocaine	12 (20)	123 (6)	135 (6)	<0.001^1^	0.59
Adrenaline dose (mg), mean ± SD	5.63 ± 12.52	2.26 ± 4.79	2.36 ± 5.18	<0.001^3^	0.65
Number of defibrillation attempts, mean ± SD	0.49 ± 0.70	0.94 ± 0.55	0.44 ± 0.55	0.800^3^	0.10
Tracheal intubation, n (%)	31 (51)	721 (33)	752 (34)	0.004^1^	0.37
Artificial respiration, n (%)	43 (70)	932 (43)	975 (44)	<0.001^1^	0.56
Hypothermia therapy, n (%)	4 (7)	29 (1)	33 (1)	0.011^2^	0.43

OHCA, out-of-hospital cardiac arrest; ROSC, return of spontaneous circulation; SD, standard deviation. ^1^Chi-square test. ^2^Fisher’s exact test. ^3^Mann-Whitney test.

### Comparing rates of survival to discharge between the two groups

We performed a retrospective analysis of statistical power using the number of study subjects (hydrocortisone group, n = 61 and non-hydrocortisone group, n = 2172) and the incidence of outcomes (hydrocortisone group, 21% and non-hydrocortisone group, 11%) by setting the α error to 0.05: The study’s statistical power was calculated to be 63%^12^. The crude odds ratio (OR) of survival to discharge was 2.2 (95% confidence interval [CI]: 1.12–3.97, *p* = 0.015) in the hydrocortisone relative to the non-hydrocortisone group. After adjusting for baseline characteristics using the inverse probability of treatment weighting (IPTW) method (Table 2), the adjusted OR was 4.2 (95% CI: 1.60–10.98, *p* = 0.004) (Table 3). In addition, we used the IPTW method to analyze the data of the 1817 cases whose dates of hydrocortisone administration and cardiac arrest we were able to determine. The hydrocortisone group tended to have a higher rate of survival to discharge than the non-hydrocortisone group (22% [10/46 cases] vs. 12% [214/1771 cases], respectively), but not significantly so (OR: 3.43, 95% CI: 0.88–13.44, *p* = 0.077). Next, we extracted a 1:1 matched cohort using the propensity score matching method (Table 2): 48 cases were matched. Although the rate of survival to discharge tended to be higher in the hydrocortisone group (OR: 2.8, 95% CI: 0.88–8.64, *p* = 0.083), there was no statistically significant difference (Table 3).

**Table 2:** Weighted and matched baseline characteristics of patients in the hydrocortisone and non-hydrocortisone groups.

	IPTW cohort			Matched cohort		
	Hydrocortisone group (n = 61)	Non-hydrocortisone group (n = 2172)	Standardized mean difference	Hydrocortisone group (n = 48)	Non-hydrocortisone group (n = 48)	Standardized mean difference
Age (years), mean ± SD	51.92 ± 14.23	51.27 ± 13.19	0.05	51.65 ± 14.68	49.65 ± 14.94	0.14
Male sex, n (%)	40 (65)	1586 (73)	0.18	32 (67)	26 (54)	0.26
OHCA, n (%)	13 (22)	326 (15)	0.19	5 (10)	5 (10)	<0.01
ROSC, n (%)	5 (8)	195 (9)	0.01	9 (19)	13 (27)	0.20
Comorbidity, n (%)						
Ischaemic heart disease	15 (24)	760 (35)	0.22	14 (29)	14 (29)	<0.01
Heart failure	14 (23)	782 (36)	0.28	15 (31)	16 (33)	0.04
Chronic lung disease	17 (28)	586 (27)	0.03	20 (42)	24 (50)	0.17
Hypertension	29 (47)	1260 (58)	0.23	28 (58)	26 (54)	0.08
Diabetes	20 (32)	956 (44)	0.24	20 (42)	19 (40)	0.04
Cerebrovascular disease	15 (25)	456 (21)	0.09	9 (19)	7 (15)	0.11
Renal disease	7 (11)	304 (14)	0.09	12 (25)	9 (19)	0.15
Liver disease	15 (24)	652 (30)	0.13	21 (44)	12 (25)	0.40
Adrenal disease	0 (0)	22 (1)	0.09	0 (0)	0 (0)	<0.01
Hyperlipidaemia	16 (27)	586 (27)	0.01	13 (27)	11 (23)	0.10
Cancer	27 (44)	847 (39)	0.10	28 (58)	26 (54)	0.08
Drugs administered, n (%)						
Vasopressin	1 (1)	22 (1)	0.02	1 (2)	1 (2)	<0.01
Methylprednisolone	2 (4)	109 (5)	0.02	7 (15)	4 (8)	0.20
Dopamine	23 (38)	500 (23)	0.36	28 (58)	30 (62)	0.08
Noradrenaline	12 (19)	326 (15)	0.13	17 (35)	18 (38)	0.04
Amiodarone	5 (9)	109 (5)	0.19	4 (8)	2 (4)	0.17
Nifecarant	1 (1)	22 (1)	0.08	1 (2)	2 (4)	0.12
Lidocaine	4 (7)	130 (6)	0.03	10 (20)	3 (6)	0.13
Adrenaline dose (mg), mean ± SD	2.82 ± 6.341	2.56 ± 7.14	0.04	4.48 ± 12.62	2.00 ± 2.98	0.27
Number of defibrillation attempts, mean ± SD	0.29 ± 0.48	0.44 ± 0.55	0.26	0.40 ± 0.54	0.38 ± 0.49	0.04
Tracheal intubation, n (%)	32 (53)	130 (34)	0.41	24 (50)	16 (33)	0.34
Artificial respiration, n (%)	34 (55)	325 (44)	0.22	33 (69)	28 (58)	0.22
Hypothermia therapy, n (%)	1 (2)	22 (1)	0.03	1 (2)	3 (6)	0.21

OHCA, out-of-hospital cardiac arrest; ROSC, return of spontaneous circulation; SD, standard deviation.

**Table 3:** Crude and adjusted odds ratios for survival to discharge (hydrocortisone group relative to the non-hydrocortisone group)

	Crude			IPTW cohort			Matched cohort		
	OR	95% CI	*p*-value	OR	95% CI	*p*-value	OR	95% CI	*p*-value
Hydrocortisone group	2.2	1.12–3.97	0.015	4.2	1.60–10.98	0.004	2.8	0.88–8.64	0.083

IPTW, inverse probability of treatment weighted; OR, odds ratio; CI, confidence interval.

### Length of hospital stay

**Figure 1:**
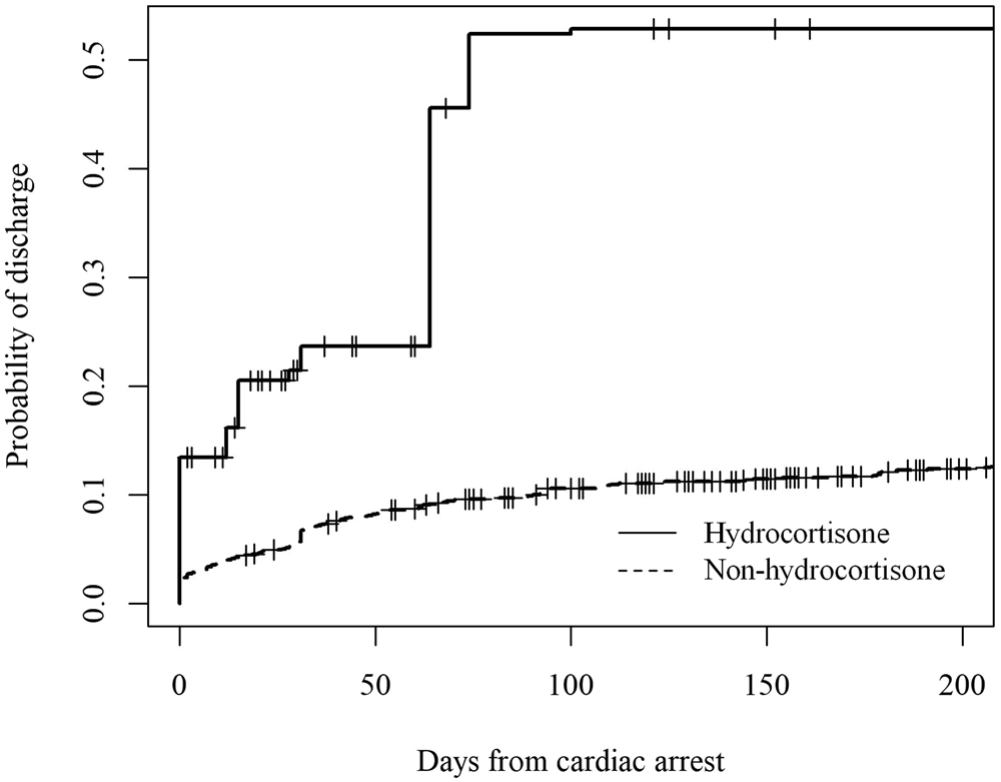
Probability of survival to hospital discharge, after adjustment using the IPTW method. The cumulative rate of survival to discharge adjusted for age of the hydrocortisone group is high in any given point. IPTW, inverse probability of treatment weighting.

The median length of hospital stay from the time of cardiac arrest of hydrocortisone group and non-hydrocortisone group were 15 days (interquartile range: 0–47.5) and 31 days (interquartile range: 3–122), respectively. In the Cox proportional hazard regression analysis, adjusted using IPTW, age (hazard ratio: 1.02, 95% CI: 1.01–1.03, *p* = 0.003) and administration of hydrocortisone (hazard ratio: 4.61, 95% CI: 2.18–9.72, *p* < 0.001) were associated with a higher rate of survival to discharge (Fig. 1, Table 4).

**Table 4:** Hazard Ratios for survival to discharge adjusted by IPTW.

	Hazard ratio	95% CI	*p*-value
Age	1.0	1.01-1.03	0.003
Administration of hydrocortisone	4.6	2.18–9.72	<0.001

IPTW, inverse probability of treatment weighted; CI, confidence interval.

## Discussion

The purpose of the present study was to assess the effect of hydrocortisone on the outcome of patients with cardiac arrest; we demonstrated an association between hydrocortisone administration and high rates of survival to discharge. Although the effectiveness of glucocorticoid administration during and after resuscitation has been reported^7–9^, the effectiveness of hydrocortisone administration has been inconclusive. Tsai *et al*. investigated whether outcomes improved when hydrocortisone 100 mg was administered during the resuscitation of OHCA patients^10^. They found that ROSC rates improved, but that there was no significant difference in survival discharge rate. Donnino *et al*. conducted a randomized, double-blind, placebo-controlled study^11^. Either hydrocortisone 100 mg or placebo was administered to study subjects for seven days or every eight hours until shock reversal. The result indicated that there was no significant difference in shock reversal or survival discharge rates.

In the present study, unlike previous reports, hydrocortisone administration after cardiac arrest was associated with higher rates of survival to discharge. One of the differences between the present study and previous studies is the number of in-hospital cardiac arrest (IHCA) patients included. Indeed, the study by Tsai *et al*. was limited to OHCA patients and in the study by Donnino *et al*., 76% of patients had OHCA. However, the present study sample comprised 85% IHCA patients. Therefore, one of the major features of our research is that we included many patients with cancer.

In this study, the ratio of vasoactive medication use was higher in the hydrocortisone group than in the non-hydrocortisone group. Because hydrocortisone is known to have a vasopressor effect^13^, we presume that it was used to improve prognosis in patients who remained hypotensive despite the use of other vasopressors. In this study, after adjusting the balance of each factors by propensity score analysis, the odds ratios for survival to discharge was significantly higher in that group than in the non-hydrocortisone group.

We also conducted a comparison using the Cox proportional-hazards regression analyses for time from cardiac arrest to discharge: The hydrocortisone group was more likely to discharge in early. In the study by Donnino *et al*., there was no significant difference in the time to shock reversal according to hydrocortisone administration^11^. Further investigation of possibilities other than stabilization of circulatory dynamics, such as post-resuscitation encephalopathy, is necessary to elucidate the mechanism whereby hydrocortisone administration shortens the duration of hospitalization.

We acknowledge that there are several limitations to this investigation. First, the claims dataset included data with no mention of the date of cardiac arrest; these data were missing in 19% of cases. When the analysis was performed using the data that had the date of cardiac arrest, the rate of survival to discharge tended to be higher in the hydrocortisone group, but not significantly so. Second, the claims data do not include several information. For example, results such as the Cerebral Performance Category (CPC) score and laboratory test results; hence, we could not evaluate the effect of hydrocortisone on neurological outcomes. Also, information on the interval from cardiac arrest to initiating advanced life support and the quality of cardiopulmonary resuscitation provided cannot be obtained from claims data; there is a possibility that such information may influence the findings. Moreover, we could not obtain information regarding the actual indication for hydrocortisone. We are currently conducting multicentre, retrospective research—we will consider the influence of these factors in that study. Third, there is no detailed description on the timing of drug administration in the claims data used in this study. Therefore, it was difficult to examine their use separately during CPR and after ROSC, as was done in the study by Mentzelopoulos *et al*.^7,8^.

An advantage of the present study is that the study population comprised mostly IHCA patients. To date, one randomized controlled trial and one non-randomized prospective study have examined the effects of using hydrocortisone monotherapy^10,11^. One included OHCA patients only, the other comprised mainly OHCA patients. On the other hand, combination regimens including vasopressin and epinephrine are used in research involving IHCA patients^7,8^. Therefore, the effect of steroid monotherapy on the outcomes of IHCA patients is inconclusive. Indeed, in the 2015 American Heart Association Guidelines Update for Cardiopulmonary Resuscitation and Emergency Cardiovascular Care^14^, the use of steroids alone in IHCA patients is not recommended. This is because in previous studies of IHCA, steroids were used in combination with vasopressin and epinephrine^7,8^. In present study, we adjusted for the use of vasopressin and epinephrine and a propensity score analysis was performed so that the effect of hydrocortisone could be evaluated. Based on these points, the novelty of this study is that administration of hydrocortisone was associated with a high rate of survival to discharge in a patient population comprised mostly of IHCA patients, and the combined use of vasopressin and inotropes was limited.

Because of the unpredictability and urgency of cardiac arrest, it is difficult to assess the effect of medicines on the outcomes of cardiac arrest patients. Although the statistical power of this study was not high, in general, analyses using claim data from various medical facilities and patient groups is considered useful because it is easy to gather large numbers of cases. This investigation suggests the association between hydrocortisone administration and the high rates of survival to discharge. However, further research is necessary to clarify this effect, to evaluate differences in effect according to the characteristic of patients, and to determine which patients would derive the greatest benefit.

## Methods

### Data source

The health-insurance claims dataset used in this study, owned by the JMDC, includes claims submitted by medical institutions and pharmacies since January 2005 for people aged < 75 years^15^. In 2015, this database included approximately 3 million people, approximating 2% of the Japanese population^15^. Each patient is assigned a unique identifier, allowing patients to be tracked across multiple medical institutions and pharmacies. Diagnoses are described using the International Statistical Classification of Diseases and Related Health Problems, revision 10 (ICD-10) codes and codes of injuries and diseases. Medications are described using the Anatomical Therapeutic Chemical Classification System (ATC) codes and generic names. Medical actions are described using Japan-specific medical action codes and the medical fee point’s quick reference table code. Because health insurance claims are submitted together once a month, the JMDC’s claims dataset include data that do not mention the actual day of an event occurring. There was no description of the exact date of cardiac arrest in 51% (n = 1099) of cases. However, since information on the start date of medical treatment for each disease can be obtained, we determined the event date using that information. Nevertheless, we were unable to determine the exact date of cardiac arrest in 416 cases; for these patients, we used the first day of the month of their admission as the event date.

### Study population

**Figure 2:**
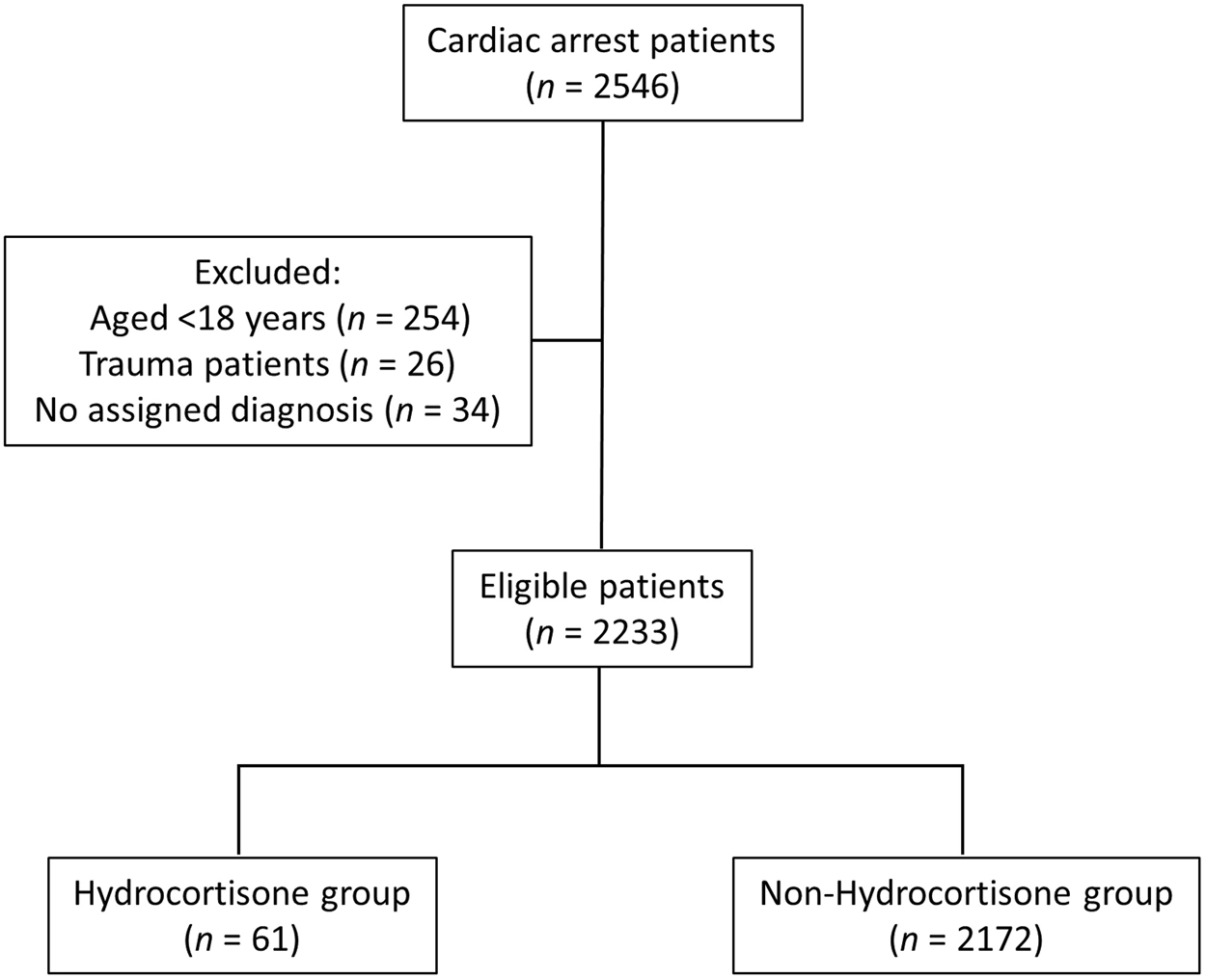
Study inclusion diagram.

In this investigation, we used the claims dataset for the period January 2005 to May 2014. Cardiac arrest was defined as a composite of cardiac arrest, paroxysmal ventricular fibrillation, pulseless ventricular tachycardia, or administration of electrical defibrillation or chest compressions. ICD-10 codes, codes of injuries and diseases, Japan-specific medical action codes, and the medical fee point’s quick reference table code were used to extract the data of cardiac arrest patients, as shown in Tables 5 and 6. Of the 2,546 patients with cardiac arrest, patients who sustained trauma, had no assigned diagnosis, or were < 18 years old were excluded, leaving 2,328 patients included for analysis (Fig. 2). These patients were categorized into 2 groups: A hydrocortisone group (patients treated with > 100 mg/day hydrocortisone sodium succinate or hydrocortisone sodium phosphate within 1 month after experiencing cardiac arrest) and a non-hydrocortisone group.

**Table 5:** Disease-related codes used to identify cardiac arrest patients.

Clinical condition	ICD-10 code	Injury and disease code
Cardiac arrest	I469	4275002
Paroxysmal ventricular fibrillation	I490	4274001
Ventricular fibrillation	I490	4274004
Pulseless ventricular tachycardia	I472	8847822

ICD-10, International Statistical Classification of Diseases and Related Health Problems, revision 10.

**Table 6:** Treatment-related codes used to identify cardiac arrest patients.

Treatment applied	Japan-specific medical action code	Medical fee point’s quick reference table code
Defibrillation	140051410 or 140010310	J047
Chest compressions	140010210	J046

### Outcome

Patients were considered to have been discharged when “the fee for providing treatment information at discharge” was assessed.

### Baseline characteristics

Cardiac arrest was defined using either ICD-10 codes (I490: ventricular fibrillation and flutter, I472: ventricular tachycardia, I469: cardiac arrest, cause unspecified) or the Japan specific medical action code (J472: countershock, J046: external cardiac massage). OHCA was defined as case that the standard disease name was out-of-hospital cardiac arrest. The definitions of medical history, medical action, and medicine are shown in Table S1.

### Statistical analysis

To describe the baseline characteristics of patients, continuous variables were summarized as the mean ± standard deviation (SD) or median and interquartile range, and categorical variables were summarized using frequencies and percentages. To compare the hydrocortisone and non-hydrocortisone groups, the Mann-Whitney U test, chi-square test, or Fisher’s exact test were performed, as appropriate (Table 1). To compare rates of survival to discharge between the two groups, simple logistic regression was performed—crude OR and 95% confidence intervals were calculated (Table 3).

A propensity score for administering hydrocortisone was calculated using multiple logistic regression. The variables used for the calculation were all factor that listed in Table 2. Propensity scores were used directly as inverse weights in estimates of the average effect, known as IPTW. Taking into account differences in patient distribution between the two groups, the baseline characteristics of each group were adjusted using the IPTW method, which allowed standardization of these characteristics to those of whole patient cohort^16^ (Table 2). The standardized mean difference was used to assess the balance of baseline covariates before and after applying IPTW; this difference should be < 0.25^17^. A weighted logistic regression analysis, using IPTW, was performed to estimate the OR comparing the rates of survival to discharge between the two groups (Table 3). Similarly, we used the IPTW method to analyze the data of the 1,817 cases whose dates of hydrocortisone administration and cardiac arrest were known. As confirmation, we used the propensity score matching method to balance the distribution of covariates between the two groups: The hydrocortisone group and non-hydrocortisone groups were matched 1:1 using nearest neighbor matching. Each matching pair had a propensity score that differed by < 0.001.

A weighted Cox proportional hazards regression analysis, using IPTW, was performed to create survival curves and to estimate the hazard ratio and 95% CI of survival to discharge. Because age is a well-known confounder that has a marked effect on survival to discharge, this variable was included as a covariate in the Cox proportional hazards model. The results are shown in Table 4 and Fig. 1. Patients were censored if they died or discontinued their insurance cover.

Analyses were performed using R statistical software version 3.3.2., and statistical significance was defined as a *p*-value < 0.05.

### Ethics statement

This study was conducted in keeping with the Ministry of Health, Labour, and Welfare’s Ethical Guidelines for Epidemiological Research^18^. It was approved by the Okayama University Graduate School of Medicine, Dentistry, and Pharmaceutical Sciences and Okayama University Hospital Ethics Committee (No. 105056), and conformed to the tenets of the Declaration of Helsinki. Since this study was an observational study with anonymized information, with no treatment intervention and no collection of human samples, obtainment of informed consent was exempted.

### Availability of data and materials

All data generated or analysed during this study are included in this published article.

## Electronic supplementary material

supplementary_table_1
