# Supplementary material for: Hydrocortisone administration was associated with improved survival in Japanese patients with cardiac arrest
Source: Sci Rep. 2017 Dec 20;7:17919. doi: 10.1038/s41598-017-17686-3 (PMC5738407; doi:10.1038/s41598-017-17686-3)
Supplement: Supplementary file 1 — supplementary_table_1 [file 41598_2017_17686_MOESM1_ESM.doc]

**Hydrocortisone administration was associated with improved survival in Japanese patients with cardiac arrest**

Takahiro Niimura^1, 3^, Yoshito Zamami^1,2,3,*^, Toshihiro Koyama^4,*^, Yuki Izawa-Ishizawa^5^, Tadashi Koga^6^, Keisaku Harada^7^, Masashi Miyake^3^, Ayako Ohshima^8^, Toru Imai^9^, Yutaka Kondo^10^, Masaki Imanishi^1,2^, Kenshi Takechi^11^, Keijo Fukushima^12^, Yuya Horinouchi^5^, Yasumasa Ikeda^5^, Hiromichi Fujino^12^, Koichiro Tsuchiya^13^, Toshiaki Tamaki^5^, Shiro Hinotsu^14^, Mitsunobu R. Kano^8^, Keisuke Ishizawa^1,2^

1. Department of Clinical Pharmacology and Therapeutics, Institute of Biomedical Sciences, Tokushima University Graduate School, 3-18-15 Kuramoto, Tokushima, 770-8503 Japan.

2. Department of Pharmacy, Tokushima University Hospital, 2-50-1 kuramoto-cho, Tokushima, 770-8503 Japan.

3. Department of Emergency Pharmaceutical Science, Graduate School of Medicine, Dentistry and Pharmaceutical Sciences, Okayama University, 1-1-1 Tsushima-naka, Okayama 700-8530 Japan.

4. Department of Clinical Pharmacy, Graduate School of Medicine, Dentistry and Pharmaceutical Sciences, Okayama University , 2-5-1 Shikata-cho, Kita-ku, Okayama, 700-8558 Japan.

5. Department of Pharmacology, Institute of Biomedical Sciences, Tokushima University Graduate School, 3-18-15 Kuramoto, Tokushima, 770-8503 Japan.

6. Drug Safety Research Laboratories, Shin Nippon Biomedical Laboratories, Ltd, 2438

Miyanoura Kagoshima, 891-1394, Japan.

7.Department of Pharmacy, Kitakyushu City Yahata Hospital 4-18-1 Nishihonmachi, Yahatahigashi-ku, Kitakyushu-shi, Fukuoka, 805-8534 Japan.

8. Department of Pharmaceutical Biomedicine, Graduate School of Medicine, Dentistry and Pharmaceutical Sciences, Okayama University, 1-1-1 Tsushima-naka, Kita-ku, Okayama-shi, Okayama, 700-8530 Japan.

9. Department of Pharmacy, Nihon University Itabashi Hospital, 30-1 Oyaguchi-Kami Machi, Itabashi-ku, Tokyo, 173-8610 Japan.

10. Department of Surgery, Beth Israel Deaconess Medical Center, Harvard Medical School, 330, Brookline Avenue, Boston, MA 02215 USA

11. Clinical Trial Center for Developmental Therapeutics, Tokushima University Hospital, 2-50-1 Kuramoto-cho, Tokushima, 770-8503 Japan.

12. Department of Molecular Pharmacology, Faculty of Pharmaceutical Sciences & Institute of Biomedical Sciences, Graduate School, Tokushima University, 1-78-1 Shinkura-cho, Tokushima, 770-8501 Japan.

13. Department of Medical Pharmacology, Institute of Biomedical Sciences, Tokushima University Graduate School, 1-78-1 Shinkura-cho, Tokushima, 770-8501 Japan.

14. Center for Innovative Clinical Medicine, Okayama University Hospital, 2-5-1 Shikata-cho, Kita-ku, Okayama, 700-8558 Japan.

*To whom correspondence may be addressed. Email: zamami@tokushima-u.ac.jp or [koyam-t@cc.okayama-u.ac.jp](mailto:koyam-t@cc.okayama-u.ac.jp)

**Supplementary Table S1.** Codes used to identify cardiac arrest patients, dugs administered, and treatment provided

|  | ICD-10 code | Medical fee point’s quick reference table code or Japan-specific medical action codes | Drug's generic name |
| --- | --- | --- | --- |
| **Status** |  |  |  |
| OHCA |  | A300 |  |
| ROSC | I460 |  |  |
| **Comorbidity** |  |  |  |
| Ischaemic heart disease | I20 – I25 |  |  |
| Cardiac failure | I50 |  |  |
| Chronic lung disease | J40 – J47 |  |  |
| Hypertension | I10 – I15 |  |  |
| Diabetes | E10 – E14 |  |  |
| Cerebrovascular disease | I60 – I69 |  |  |
| Renal disease | N00 – N08, N10 – N19 |  |  |
| Liver disease | K00 – K77 |  |  |
| Adrenal disease | E25, E27 |  |  |
| Hyperlipidaemia | E785 |  |  |
| Cancer | C00 – D48 |  |  |
| **Drugs administered** |  |  |  |
| Vasopressin |  |  | "Vasopressin"(over 20 Units/day) |
| Methylprednisolone |  |  | "Methylprednisolone succinate" or " Methylprednisolone acetate"(over 40 mg/day) |
| Dopamine |  |  | "Dopamine hydrochloride" |
| Noradrenaline |  |  | "Noradrenaline" |
| Amiodarone |  |  | "Amiodarone hydrochloride" |
| Nifecarant |  |  | "Nifekalant hydrochloride" |
| Lidocaine |  |  | "Lidocaine hydrochloride" or |
| Adrenaline dose |  |  | "Adrenaline"(Total dose on cardiac arrest date) |
| **Treatment** |  |  |  |
| Number of defibrillation attempts |  | J047 (Total number on cardiac arrest) |  |
| Tracheal intubation for life-saving |  | J044 |  |
| Artificial respiration |  | J085 |  |
| Hypothermia therapy |  | L008 - 2 |  |
| **Outcome** |  |  |  |
| the fee for providing treatment information at discharge |  | B009 |  |
| Tracheal intubation | Z991 | 140006050, 140009310, 140023510, 140030830, 140039550, 140039650, 114006810, 114009610, 190120970, 190120970, 190055470, 999900022 |  |

ICD-10, International Statistical Classification of Diseases and Related Health Problems, revision 10.
